# Discrimination against HIV/AIDS patients and associated factors among women in East African countries: using the most recent DHS data (2015–2022)

**DOI:** 10.1186/s41043-023-00491-2

**Published:** 2024-01-02

**Authors:** Bewuketu Terefe, Mahlet Moges Jembere

**Affiliations:** 1 Department of Community Health Nursing, School of Nursing, College of Medicine and Health Sciences, University of Gondar, Post Box 196, Gondar, Ethiopia; 2 Department of Emergency, and Critical Care Nursing, School of Nursing, College of Medicine and Health Sciences, University of Gondar, Gondar, Ethiopia

## Abstract

**Introduction:**

The biggest health problem in East Africa is the human immunodeficiency virus (HIV). Combating stigma and discrimination related to HIV/AIDS is a key goal of many international organizations in their efforts to ensure universal access to HIV/AIDS prevention, treatment, care, and support programs. However, previous studies in various regions of Africa have shown that the prevalence of discriminatory attitudes related to HIV/AIDS is particularly high. Furthermore, there is a current evidence gap in the region. Therefore, the aim of this study was to determine the prevalence of discriminatory attitude toward HIV/AIDS patients, and its associated factors among women in East African countries.

**Methods:**

The data we utilized were gathered from the most recent Demographic and Health Surveys (DHS), which were carried out in east African nations between 2016 and 2022. We integrated DHS data from ten countries into our investigation. For our analysis, a weighted sample of 139,812 women overall was employed. The analysis used multiple logistic regressions. The adjusted odds ratio and its 95% confidence interval were then shown, and components with binary logistic regression p values of less than or equal to 0.2 and < 0.05 were regarded as significant predictors of discrimination against HIV/AIDS patients.

**Results:**

In this study, 32.73% (95% CI 34.48–32.97) of respondents had a discriminatory attitude toward HIV/AIDS patients. In the multiple logistic regression analysis, being in the older age groups, having a better education level, being from a wealthy household, having employment status, having ANC follow-up, institutional delivery, mass media exposure, and having female household heads were associated with higher odds of not having a discriminatory attitude toward HIV/AIDS patients. However, being unmarried and living far from the health facilities were associated with higher odds of discriminatory attitudes toward HIV/AIDS patients.

**Conclusion:**

This study concluded that women in East Africa still had a very discriminatory attitude toward HIV/AIDS patients. The good news for East Africa is that prevalence has decreased when compared to earlier findings. Improving women's empowerment, maternal health services, and health facilities' accessibility are crucial.

## Introduction

Stigma and discrimination around HIV continue to put those who are infected at risk and prevent millions of people from accessing services for testing, prevention, and treatment [1, 2]. These issues pose serious obstacles to reaching international agreements aimed at ending the HIV pandemic. Numerous research have discovered a link between HIV-related stigma and low participation in biological preventive measures, non-disclosure to partners, and unwillingness to test for the virus [3–7]. In addition, stigma and discrimination impact individuals living with HIV in emergency and humanitarian situations, the legal system, the workplace, families, and communities [8, 9].

By the year 2020, there were over 37.6 million people living with HIV worldwide, and every week, about 5000 young women between the ages of 15 and 24 contract the virus [10]. Sub-Saharan Africa (SSA) currently bears a disproportionately large share of the cost of the global HIV epidemic; there, 71% of people with HIV live [11, 12]. Moreover, there were approximately 75% deaths and 65% new infections in 2017. The HIV epidemic continues to be most severe in Eastern and Southern Africa, where 53% of people living with HIV worldwide and 45% of all new HIV infections occur. Shocking reductions in HIV infections and AIDS-related deaths are being brought about by a strong sense of shared responsibility among the region's governments, civil society, international funders, and the research community [13]. Goal 3 of the Sustainable Development Agenda asks for the eradication of the HIV/AIDS pandemic by 2030 in order to halt and reverse its spread. Furthermore, by 2030, the Joint United Nations Program on HIV/AIDS (UNAIDS) aims to reduce both new infections and fatalities. Despite these objectives, a recent assessment of the HIV situation found that the HIV epidemic has not yet been curbed globally [14–16].

One of the reported barriers to achieving universal access to HIV/AIDS prevention, treatment, care, and support programs is differential action or behavior toward the stigmatized person based on those attitudes and perceptions in developing countries where strong cultural, moral, and religious values are highly practiced throughout communities [17, 18]. Due to the nature of HIV/AIDS, which is due to its fatality, contagiousness, and transmissibility, as well as the repellent, ugly, and upsetting appearance of the infected individual in the advanced stages of the disease, as well as its mode of transmission, transmitted through sexual intercourse that is perceived as a result of sexual immoral behaviors, people living with HIV are severely discriminated against [19, 20].

The negative effects of stigma and discrimination within communities and families include medication non-adherence, avoidance or delaying of necessary care and treatment, increased psychological distress, physical and emotional/verbal abuse, a lack of social support, isolation, and risky health behaviors like medication hiding [21, 22]. Additionally, stigma and prejudice are blamed for being key obstacles to HIV prevention and care program success [18]. As a result, those who are at risk of contracting HIV or are unsure if they do may decide against getting tested for the virus out of concern for stigma and out of concern for maintaining their privacy and confidentiality regarding their HIV status in medical settings [23, 24]. The prevalence of discriminatory attitudes connected to HIV/AIDS has previously been shown to range from 40% to 93.8% in various regions of Africa [25–27].

Previous studies have identified a number of significant factors that influence discriminatory attitudes toward PLWHA, including education level, financial situation, employment status, internet use, place of residence, media exposure, HIV testing, marital status, region, high-risk behavior, people with stigmatized identities, HIV infection sources, disease stage, and relationships with infected people [18, 27–29].

Interventions to lessen discriminatory attitudes are necessary to combat HIV/AIDS transmission and improve the quality of life for people living with HIV due to the aforementioned detrimental effects of discriminatory attitudes. Even if there are many PLWH in East Africa, the causes of discriminatory attitudes toward PLWH have not been adequately addressed. The few accessible studies were conducted locally, but none of them were covered large area in scope. This study makes use of nationally representative data that may be applied to all women in East Africa who are of reproductive age. No matter the respondents' gender, past research has been conducted among all adults aged 15 to 49. However, the study did not address the question of what factors cause reproductive age women to see PLWH with discrimination. Therefore, using data from the recent East African surveys, this study sought to determine the prevalence, and uncover the factors that contribute to women's discriminatory views about PLWH among those of reproductive age.

## Methods

### Study setting, and period

Data were collected in East African countries between 2015 and 2022 for a cross-sectional study design that was conducted nationwide in communities from Burundi, Ethiopia, Kenya, Madagascar, Malawi, Rwanda, Uganda, Zambia, and Zimbabwe. The United States Agency for International Development (USAID) funds the Demographic and Health Surveys (DHS) Program, which provides funding and technical support for population and health surveys in countries all over the world. This support was provided by ICF. The most recent DHS data set for East African nations during the last five-year period (2015–2022) provided the information for this study. A standardized data set was employed [30] to collect a sizable sample size that is representative of the population source and all factors. DHS gathers comparable data on a global scale. The surveys have huge sample sizes, are population-based, and nationally representative of each nation [30]. The 14 nations that make up Eastern Africa are spread throughout the Horn of Africa, the Indian Ocean islands, and the Great Lakes region. These nations struggle with comparable economic, social, and environmental problems, and they worry that they would not achieve all of the Millennium Development Goals' objectives [31]. East Africa is the portion of the African continent that lies in the horn and Eastern parts of the Sahara Desert. They are estimated to be home to 486,766,759 people and cover an area of 6,667,493 km^2^ (2,574,332 square miles), making up 6.03% of the world's population.

### Data source and study population

We used DHS that were completed throughout the last five years, from 2015 to 2022. Although approximately 14 countries in the east undertook DHS between 2015 and 2022, only roughly nine countries' DHS were utilized for this study since the remaining countries' surveys lacked information on the outcome variable. A total weighted sample of 139,812 women and a total unweighted sample of 139,593 women who had ever heard of AIDS were utilized for the final analysis once each country's data had been added (Table 1).

**Table 1:** Countries, sample size, and survey year of demographic and health surveys included in the analysis for nine East African countries

Country	Survey year	Sample size(weighted)	Frequency(weighted)
Burundi	2016/17	16,308	11.66
Ethiopia	2016	14,035	10.04
Kenya	2022	16,207	11.59
Madagascar	2021	14,364	10.27
Malawi	2016/17	23,698	16.97
Rwanda	2019/20	14,542	10.40
Uganda	2016	18,076	12.93
Zambia	2018	12,868	9.20
Zimbabwe	2015	9714	6.95

### Sample size determination and sampling procedures

Demographic and health survey reports were available for around 12 of the 13 East African nations. Every five years, a systematic collection of DHS surveys is conducted in low- and middle-income nations using pretested, validated, and structured questionnaires. Multi-country analysis is possible since the DHS surveys use the same standard approach for sampling, questionnaires, data collection, and coding. The most recent conventional census frame was used in each of the surveys carried out in the nations indicated. DHS samples are frequently divided between urban and rural areas within each administrative geographic region. A stratified two-stage cluster sampling technique is used in the DHS survey. Clusters and enumeration areas (EAs), which are typically created from the most recent national census available, were randomly chosen from the sample frame in the first stage. On the households mentioned in each cluster or EA, systematic sampling was used in the second step. In the first round of sampling, enumeration areas (EAs) were selected with a probability proportional to the size of each stratum. The systematic sampling approach chooses a predetermined number of households in designated EAs in the second step of sampling. Following the listing of the households, equal probability systematic sampling is used to select a certain number of households inside the defined cluster [30].

### Data quality control

In the DHS, a pretest was conducted before data collection, a debriefing session with pretest field workers was held, and changes to the questionnaires were made as necessary. The DHS guidance provides additional details regarding the data-collection process. Details can be accessed from the Guide to DHS statistics.

### Data processing and statistical analysis

The standard DHS data set was downloaded in STATA format before being cleaned, integrated, transformed, and appended to provide useful variables for the analysis. To define variables in the study using statistical measurements, Microsoft Excel 2019 and STATA version 17 software were used to obtain both descriptive and analytic statistics [32]. Variables with a p value of 0.2 or below in the binary analysis were considered for the multiple analysis. We looked at the data under the assumption of a multilevel model analysis using the intra-class correlation (ICC) coefficient because the data might be hierarchical, but since it was less than 5%, it did not fulfill the basic need to conduct it. Thus, it was shown that traditional logistic regression was more effective. To identify the factors that are linked with HIV discriminatory attitude, a multivariable logistic model was used. The model's adjusted odds ratio (AOR) with 95% confidence interval (CI) was presented. Descriptive analyses, such as frequency count and proportion for categorical data, were utilized to summarize the descriptive data. Bivariable logistic regression was used to choose potential variables for multiple logistic regression. A logistic model was fitted to test for multicollinearity among the independent variables using the variance inflation factor. The overall fitness of the final regression model was further assessed using the Hosmer and Lemeshow test. The statistical significance level for the final model was set at p value of less than 0.05. Before proceeding to the analysis, each dependent variable was assessed for its variance, inflation factors, and tolerances. The mean VIF in this study was 1.05.

## Variables of the study

### The outcome variable

The outcome variable of this study was the percentage of women who have discriminatory attitudes toward people living with HIV among women who have heard of HIV or AIDS. The definition of the outcome variable (discriminatory attitude toward people living with HIV/AIDS) was defined as “among women who have heard of HIV or AIDS: 1) percentage of women who do not think that children living with HIV should be able to attend school with children who are HIV negative, and 2) percentage of women who would not buy fresh vegetables from a shopkeeper who has HIV.” Then, the number of women who respond “no” to either of the above two questions is said to be because they have discriminatory attitudes toward people living with HIV. The outcome variable of this study was the percentage of women who have discriminatory attitudes toward people living with HIV among women who have heard of HIV or AIDS. The definition of the outcome variable (discriminatory attitude toward people living with HIV/AIDS) was defined as “among women who have heard of HIV or AIDS: 1) percentage of women who do not think that children living with HIV should be able to attend school with children who are HIV negative, and 2) percentage of women who would not buy fresh vegetables from a shopkeeper who has HIV.” Then, the number of women who respond “no” to either of the above two questions is said to be because they have discriminatory attitudes toward people living with HIV. Then, the outcome variable was recategorized as "Yes" = “1” if the women responded “yes” to the above questions; if not, it was recoded as “no.” This classification and the analysis have been made according to the guide to the DHS statistics book. Similarly, regarding the missing values, only when a respondent selects “No” for either question is their attitude toward discrimination deemed to be present. Responses that include “Don't know/Not sure/Depends” or lack certain values are not seen as indicative of discriminatory views [30].

### The independent variables

Independent variables: Various maternal-related factors were included. All these variables were included after reviewing previous literatures based on their proximity to the outcome variable [23, 33–35]. This included maternal age (15–24, 25–34, and 35–49 years old), educational status (not educated, primary, and secondary/higher), types of places of residence (urban, rural), marital status (not married, married), household wealth index (poorest, poorer, middle, richer, and richest), current employment status (no, yes), mass media exposure (no, yes0, ANC follow-up (no, yes), place of delivery (health facility, home), number of health visits (once, more than once), visited by field workers in the past 12 months (no, yes), contraceptive utilization methods (no utilized, traditional, and modern methods), distance to the health facility (not perceived as a big problem, big problem), comprehensive knowledge of HIV/AIDS (poor, good), sex of the household head (male, female0, and breastfeeding status (no, yes) were included.

## Results

### Sociodemographic characteristics of the study participant

In this study, a total of 139,812 women of reproductive age were enrolled in east African countries. About 56,573 (40.46%) of the study women were between 15 and 24 years of reproductive age. Regarding marital status, nearly half of mothers (68,914) (49.29%) were married. With respect to place of residence types 101,727 (72.76%), educational status 64,640 (46.23%), wealth index 35,579 (25.45%), place of delivery 125,481 (989.75%), and ANC follow-up 136,072 (97.32%), the mothers were from rural areas, had primary educational status, were in the richest households, had institutional delivery, and had at least one ANC follow-up during their pregnancies, respectively. Furthermore, about 71,761 (51.33%) and 84,844 (60.68%) of women have at least one mass media exposure (either listening to radio, watching television, or reading magazines or newspapers), respectively. However, more than half (87,890 or 62.86%) and the majority (89,608 or 64.09%) of mothers did not utilize any method or type of contraceptive, and they reported that the distance to the health facility is a major problem to visit it. Similarly, about 104,607 (74.82%) of women were not visited by the health field worker throughout the solid year. Furthermore, about 110,959 (79.36%) participants had only one health facility visit per year, and only 97,026 (30.60%) of households had a female household head. With regard to knowledge on HIV/AIDS, about 22,984 (75.86%) of women have comprehensive knowledge. (Table 2).

**Table 2:** Sociodemographic and maternal-related characteristics of respondent’s discriminatory attitudes toward people living with HIV/AIDS among reproductive age women in East African countries (weighted *n* = 139,812)

Variables	Categories	Frequency	Percentage
Age in years	15–24	56,573	40.46
	25–34	43,463	31.09
	35–49	39,776	28.45
Residence	Urban	38,085	27.24
	Rural	101,727	72.76
Mothers’ educational status	No education	21,061	15.06
	Primary	64,640	46.23
	Secondary and higher	54,111	38.70
Mother is employed	No	54,968	39.32
	Yes	84,844	60.68
Wealth index	Poorest	23,147	16.56
	Poorer	25,076	17.94
	Middle	26,485	18.94
	Richer	29,526	21.12
	Richest	35,579	25.45
Mass media exposure	No	68,051	48.67
	Yes	71,761	51.33
Contraceptive methods	No method	87,890	62.86
	Traditional methods	3646	2.61
	Modern methods	48,276	34.53
Number of health visits in the past 12 months	Once	110,959	79.36
	More than one	28,853	20.64
Distance from health facility	Not a big problem	50,204	35.91
	A big problem	89,608	64.09
Visited by field worker in the past 12 months	No	118,023	84.42
	Yes	21,789	15.58
Currently breastfeeding	No	104,607	74.82
	Yes	35,205	25.18
Marital status	Not married	70,898	50.71
	Married	68,914	49.29
HIV comprehensive knowledge(*n* = 30,298)	No	7314	24.14
	Yes	22,984	75.86
ANC follow-ups	No	3740	2.68
	At least one	136,072	97.32
Place of delivery	Home	14,331	10.25
	Health facility	125,481	89.75
Sex of the household head	Male	97,026	69.40
	Female	42,786	30.60

### Factors associated with HIV discriminatory attitude with people living with HIV/AIDS among reproductive age women in east Africa

The odds of not having an HIV discriminatory attitude were increased by 65% and 67% (AOR = 1.65, 95% CI: 1.59–1.69) more times among women whose age is from 25–34 years and from 35–49 years old, respectively, as compared to women whose age is grouped from 15–24 years old. In addition to this, as compared to uneducated women, those who had accomplished primary and secondary/higher educational attainment showed (AOR = 1.87, 95% CI 1.81–1.94) and (AOR = 2.94, 95% CI 2.83–3.06) times more chances of not having HIV discriminatory attitudes for people living with HIV/AIDS, respectively. Those mothers who are currently employed have shown an 8% higher likelihood of not having a bad discriminatory attitude toward people living with HIV/AIDS (AOR = 1.08; 95% CI 1.06–1.11) when compared to unemployed mothers. Similarly, regarding household wealth index, mothers who came from poorer, middle, richer, and richest households have shown a higher likelihood of not having a bad HIV discriminatory attitude for people living with HIV/AIDS as compared to women who came from the poorest household wealth index by the odds of (AOR = 1.13, 95% CI 1.09, 1.17), (AOR = 1.19, 95% CI: 1.15, 1.24), (AOR = 1.25, 95% CI 1.46, 1.59), respectively. Women who had at least one ANC follow-up and had given birth at health institutions had an odds ratio of (AOR = 1.68, 95% CI 1.54, 1.82), and (AOR = 2.62, 95% CI 2.51, 2.72), respectively, of not being discriminatory women for HIV/AIDS patients as compared to their counterparts. Those mothers who have mass media exposure have shown 28% less of an HIV discriminatory attitude by the odds (AOR = 1.28; 95% CI 1.25–1.32) compared to their counterparts. Female household heads have shown an 18% higher likelihood of not being discriminators for HIV/AIDS patients as compared to male household heads (AOR = 1.18; 95% CI 1.15–1.21). On the other hand, regarding distance to the health facility, those mothers who have lived distant from the health facility and have reported that distance to the health facility is a big problem have shown a lower likelihood (AOR = 0.97, 95% CI 0.95, 0.99) of not being discriminatory women as compared to mothers who have reported that distance to the health facility is not a big problem for their basic health needs (Table 3).

**Table 3:** Multiple logistic regression analysis results on determinants discriminatory attitudes toward people living with HIV/AIDS among reproductive age women in East African countries (weighted *n* = 139,812, and unweighted *n* = 139,593)

HIV discriminatory attitude	No, *n* (%)	Yes, *n* (%)	COR 95% CI	AOR 95% CI
*Variables*				
Maternal age				
15–24	20,219 (35.74)	36,354 (64.26)	1	1
25–34	13,125 (30.20)	30,338 (69.80)	1.31 (1.27,1.34)	**1.65 (1.59,1.69)**
35–49	2,414 (31.21)	27,362 (68.79)	1.24 (1.21,1.28)	**1.67 (1.62,1.73)**
Maternal education				
Not educated	10,840 (51.47)	10,222 (48.53)	1	1
Primary	22,700 (35.12)	41,940 (64.88)	2.07 (2.00,2.13)	**1.87 (1.81,1.94)**
Secondary and higher	12,219 (22.58)	41,892 (77.42)	3.80 (3.67,3.93)	**2.94 (2.83,3.06)**
Employment status				
No	19,115 (34.78)	35,853 (65.22)	1	1
Yes	26,642 (31.40)	58,20 (68.60)	1.16 (1.13,1.18)	**1.08 (1.06,1.11)**
Marital status				
Married	23,253 (33.74)	45,661 (66.26)	1	1
Not married	22,505 (31.74)	48,393 (68.26)	1.09 (1.06,1.11)	**0.97 (0.94,0.99)**
Wealth index				
Poorest	9,682 (41.83)	13,465 (58.17)	1	1
Poorer	9,662 (38.53)	15,414 (61.47)	1.37 (1.33,1.43)	**1.13 (1.09,1.17)**
Middle	9,369 (35.38)	17,116 (64.62)	1.63 (1.57,1.69)	**1.19 (1.15,1.24)**
Richer	9,207 (31.18)	20,319 (68.82)	1.98 (1.91,2.06)	**1.25 (1.19,1.29)**
Richest	7,838 (22.03)	27,741 (77.97)	3.03 (2.93,3.14)	**1.52 (1.46,1.59)**
ANC follow-ups				
No	2680 (71.65)	1060 (28.35)	1	1
Yes	43,078 (31.66)	92,994 (68.34)	5.08 (4.71,5.48)	**1.68 (1.54,1.82)**
Place of delivery				
Home	8837 (61.66)	5494 (38.34)	1	1
Health facility	36,921 (29.42)	88,560 (70.58)	3.76 (3.62,3.90)	**2.62 (2.51,2.72)**
Mass media exposure				
No	27,264 (40.06)	40,787 (59.94)	1	1
Yes	18,494 (25.77)	53,268 (74.23)	1.94 (1.89,1.98)	**1.28 (1.25,1.32)**
Number of health visits				
Once	35,681 (32.16)	75,278 (67.84)	1	1
More than one	10,077 (34.92)	18,776 (65.08)	0.90 (0.87,0.92)	0.98 (0.95,1.02)
Sex of household head				
Male	33,513 (34.54)	63,513 (65.46)	1	1
Female	12,244 (28.62)	30,542 (71.38)	1.24 (1.21,1.27)	**1.18 (1.15,1.21)**
Distance to health facility				
A big problem	18,847 (37.54)	31,356 (62.46)	1.41 (1.37,1.44)	**0.97 (0.95,0.99)**
Not a big problem	26,910 (30.03)	62,698 (69.97)	1	1

Where bold numbers are significant at *P *value of less than 0.05 in the final model

### Prevalence of discriminatory attitudes among women toward HIV/AIDS patients

**Fig. 1:**
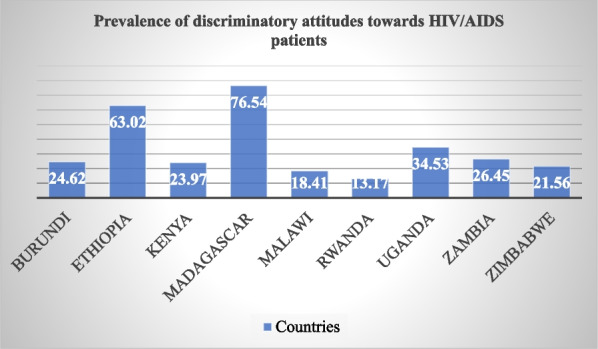
The prevalence of discriminatory attitudes toward HIV/AIDS patient across countries among reproductive age women in East Africa from 215 to 2022

The overall prevalence of discriminatory attitudes among women toward HIV/AIDS Patients in East Africa was found to be 32.73% (95% CI: 34.48, 32.97). The highest prevalence of discriminatory attitude was in Madagascar with 76.54%, and the lowest was in Rwanda with 13.17%. Uganda and Ethiopia scored more than the pooled prevalence percent in the region (Fig. 1).

## Discussion

This study examined how women of reproductive age felt about people living with HIV/AIDS and its associated factors in nine East African countries. In this study, the prevalence of prejudice against HIV/AIDS patients was 32.73% (95% CI: 34.48, 32.97). This conclusion is higher than the one in Pakistan [36], and lower than those done in SSA [33], Ethiopia [25], and Nigeria [26]. This discrepancy might be caused by a difference in the study population (since this study includes study participants in east Africa), a difference in data measurement and time, sociocultural and socioeconomic differences between countries, a difference in the study period and sample size (this study was based on pooled analysis), as well as a difference in the study period and sample size.

Compared to younger women, older women were more likely to not view those living with HIV/AIDS with discrimination. This is consistent with investigations done in Botswana and SSA [27, 33]. This might be the case because younger women are more dependent on their families and less likely to gain knowledge about HIV/AIDS earlier in life.

Regarding educational status, women with formal education were less likely to have prejudices against PLWH. This is corroborated by findings from other studies conducted in Ethiopia [28], SSA [33], Kenya [37], and Nigeria [26], where lower educational status was found to be adversely correlated with prejudice against PLWH. This may be justified by those with higher levels of education who may have access to greater information about HIV/AIDS through the media, the internet, and health services [28]. This information will enable them to provide for and have compassion for PLWH. Additionally, it could be due to the fact that education is a potent instrument that influences attitudes by encouraging a better understanding of HIV/AIDS. Additionally, educated people are more accepting of those living with HIV/AIDS and more willing to respect them. Additionally, educated people are more sympathetic toward those who are HIV/AIDS positive and more sympathetic toward sufferers' rights to interaction and survival [38].

Another factor that was linked to discrimination against people with HIV/AIDS was the household wealth index and employment status. Women from low socioeconomic/wealth status households were more likely to harbor a prejudiced attitude toward those living with HIV/AIDS. This is consistent with research done elsewhere [23, 33, 36, 39]. This may be the result of better and more relevant knowledge, higher levels of education, better access to media, and greater awareness of health issues among people from higher socioeconomic backgrounds [40].

Additionally, compared to persons who were married, those who were not married or who had a history of marriage had fewer probabilities of having a prejudiced attitude toward people who had HIV/AIDS. According to research done in Ethiopia and Nigeria, married women are more likely than unmarried women to hold a prejudiced attitude toward those living with HIV/AIDS [26, 28]. In this study, married people and those with a history of marriage may have been more likely to have a discriminatory attitude toward people living with HIV/AIDS because they learned about the disease from their spouses or because they were older and therefore more likely to have firsthand knowledge of the disease.

Related to maternal and health facility accessibility, when compared to their counterparts, women who have at least one ANC follow-up and gave birth in a hospital have more often shown themselves to be non-discriminatory toward HIV/AIDS patients; however, those who cited travel time to the hospital as a major concern have shown themselves to be more so. In SSA, a qualitative study revealed that impediments to health facility accessibility, direct and indirect costs associated with getting an HIV test, and gender inequity that compromises women's decision-making autonomy about an HIV test have all been named as major challenges [24].

Women who delivered at medical facilities and had at least one antenatal care (ANC) visit during their pregnancy will be given information about HIV/AIDS and routine opt-out HTC. Given that HIV is a growing factor in both direct and indirect causes of maternal death, there is evidence that HIV and maternal mortality are not two distinct epidemics in SSA but rather two epidemics that intersect there [41]. One of the main tactics suggested for preventing maternal mortality and morbidity is childbirth with a qualified healthcare provider. It has also been noted as crucial for improving the prevention of mother-to-child transmission (PMTCT) of HIV [42–44], by increasing women's comprehensive HIV knowledge.

Compared to male family leaders, female household heads have an 18% higher likelihood of not discriminating against HIV/AIDS patients. This demonstrates the significance of women's empowerment in combating the HIV epidemic, and discrimination against PLWHIV [45, 46]. It increases women's self-assurance to test for HIV, learn their HIV status, and be able to stop mother-to-child transmission and new infections in their partners. Access to HIV testing for women is highly correlated with a woman's level of empowerment [40]. It is thought that a woman who is empowered on all levels—culturally, politically, and professionally—has the self-assurance to determine whether to get an HIV test or not since she is not dependent on her spouse or partner to make that decision for her [40, 47].

The current study also showed that media exposure was linked to a better likelihood of not having a prejudiced attitude toward people living with HIV/AIDS. This is consistent with research from SSA [33], Pakistan [36], and Ethiopia [23]. This might be the case because effective media communication increases public awareness of HIV/AIDS. Furthermore, the media disseminates factual information, debunking damaging stereotypes and preconceived notions about HIV/AIDS. In addition, media services may help people learn more about others' experiences and alter their views of the disease and those impacted by HIV/AIDS. Additionally, through raising knowledge, encouraging positive attitudes, and promoting value systems that value kindness and caring for HIV/AIDS victims, the media can have an impact on people's attitudes and behavior around HIV/AIDS.

## Strength and limitations of the study

Concentrating on women, which are the population's most important segments, the most recent data from East Africa were used as the strength for this study, along with appropriate statistical analysis. Therefore, it can be used by policymakers, as well as governmental and non-governmental organizations, to take appropriate action. The study, however, had limitations because it was based on survey data and we were unable to consider crucial factors, including cultural norms and attitudes, and other contextual related factors toward people living with HIV/AIDS. Additionally, using variables from existing data sets is limited, as is the case with the outcome variable, which is only assessed by two questions. The cause and effect link between the outcome variable and independent variables cannot be shown because it was based on survey data. Although data collectors are well trained, highly professional, there might be interview bias due to the intentional or unintentional communication barriers. As a result, care should be taken when interpreting the study's results.

## Conclusions, and recommendations

This study concluded that women in East Africa were highly discriminatory toward PLWH. However, prevalence has revealed a reduction compared to earlier evidence, and this is good news for east Africa. Age, wealth index, education level, proximity to a health facility, female empowerment, occupation, ANC follow-up, institutional delivery, media exposure, and marital status were all statistically linked to HIV/AIDS discriminatory attitudes among women in east Africa. Therefore, individuals who are underprivileged, illiterate, or young ladies should receive special consideration. Additionally, it is preferable to improve accessibility, the proximity of medical facilities, and the availability of various media for women in order to foster a positive attitude toward those who have HIV/AIDS. By reducing discriminatory attitudes toward HIV/AIDS patients, improving maternal health care (ANC, institutional delivery, and female empowerment) will have a bigger impact on the fight against the disease. Furthermore, upcoming researchers might fill out the limitations of this study by developing tools related to cultural and contextual variables according to each country profile.

## Data Availability

All data concerning this study are accommodated and presented in this document. The detailed data set can be freely accessible from the www. dhsprogram.com website.
